# Qiangjing Tablets Regulate Apoptosis and Oxidative Stress *via* Keap/Nrf2 Pathway to Improve the Reproductive Function in Asthenospermia Rats

**DOI:** 10.3389/fphar.2021.714892

**Published:** 2021-09-06

**Authors:** Guangsen Li, Peihai Zhang, Yaodong You, Diang Chen, Jian Cai, Ziyang Ma, Xiaopeng Huang, Degui Chang

**Affiliations:** Department of Urology/Andrology, TCM Regulating Metabolic Diseases Key Laboratory of Sichuan Province, Hospital of Chengdu University of Traditional Chinese Medicine, Chengdu, China

**Keywords:** Asthenozoospermia, Keap/Nrf2, Qiangjing tablets, reproductive function, oxidative stress

## Abstract

Asthenozoospermia (AZS), is a common cause of male infertility. Currently, most drugs for azoospermia lack desirable therapeutic efficiency, therefore developing new drug therapy is important. Qiangjing tablets could enhance renal function and improve sperm quality. The purpose of this study was to examine whether Qiangjing tablets could improve the reproductive function in azoospermia rats through activating the Nrf2/ARE pathway, and how to regulate energy metabolism and oxidative stress in this process. Sperm motility, sperm concentration and sperm viability were detected by WLJY-9000 Weili Digital Color Sperm Quality Detection System. HE staining was used to observe the pathological condition of testis in AZS rats. Cell apoptosis was analyzed by Tunnel staining and flow cytometry. The changes of mitochondrial membrane potential were detected by JC-1. The levels of Estradiol, testosterone and luteinizing hormone, activity of superoxide dismutase (SOD) and glutathione peroxidase (GSH-Px), and content of malondialdehyde (MDA) and glutathione (GSH) were detected by ELISA. The effects of Qiangjing Tablets on GC-1 spgs and Nrf2 protein were investigated through CCK-8 assay and western blot. The expression levels of HO-1, Keap1, and P-Nrf2 were detected by western blot. The results demonstrated that Qiangjing tablets upregulated levels of sperm motility, sperm concentration and sperm viability, which was shown to significantly increase levels of HO-1, Keap1, P-Nrf2, Estradiol and testosterone, along with increasing the activity of SOD, GSH-Px and GSH and suppressing the MDA content, luteinizing hormone and Vimentin level. Qiangjing tablets could significantly inhibit spermatogenic cells apoptosis and promote GC-1 spgs viability, increase PE/FITC ratio, mitochondrial membrane potential and reduc oxidative stress. Qiangjing tablets protected spermatogenic cell to upregulate male sex hormoneto, improved the sperm quality and reproductive function in AZS rats via activating the Keap/Nrf2 signaling pathway.

## Introduction

Infertility is a global problem that is both social and medical (Moore and Reijo-Pera, 2000), which is calculated that the ‘male factor’ accounts for up to 50% Hwang et al. (2011) and is mainly related to sperm concentration, morphology and motility disorders. Among them asthenozoospermia (AZS), one of the most common types of male infertility (Liu et al., 2015), which can be the result of many factors (Balkan et al., 2008), for example, abnormal semen liquefaction, abnormal immunity, abnormal sperm structure, low sperm motility, deficiency of sperm energy metabolism (Tamburrino et al., 2014), abnormal signal transduction pathways (Lin et al., 2019). However, the mechanisms of azoospermia are complex and have not been defined (Du et al., 2019). Studies have assayed that ROS can contribute to sperm damage (Aitken, 1995), due to the vulnerability of sperm to oxidative attack and the occurrence of plasma membrane lipid peroxidation (Aitken et al., 1995; Gharagozloo and Aitken, 2011). Interestingly, sperm membrane lipid peroxidation reduces the mitochondrial membrane potential (DYm) (Koppers et al., 2011), Furthermore, the interrelationship between low DYm and decreased sperm viability has been reported in medical journals (Amaral and Ramalho-Santos, 2010; Zhang et al., 2016a). Changes in mitochondrial sperm membrane composition can affect the activity of the respiratory chain and oxidative phosphorylation pathways, leading to loss of energy production, and thereby lowering sperm motility (Aitken et al., 2012). Consequently, the search for antioxidant drugs for the treatment of male infertility is urgent (Kobori et al., 2014; Ajina et al., 2017).

The Nrf2-ARE pathway is an inherent mechanism of anti-oxidative defense (Petri et al., 2012). Nrf2 is involved in the anti-inflammatory process by participating in the recruitment of inflammatory cells and regulating the expression of antioxidant response element (ARE) genes. Keap1 is a Cullin3 (Cul3)-based E3-ligase bridging protein that strictly regulates the activity of Nrf2. Under regular physiological circumstances, Keap1 selectively targets Nrf2, leading to ubiquitin-dependent proteasomal degradation. When oxidative stress occurs, Keap1 inactivation allows for the inhibition of Nrf2 ubiquitination, ultimately leading to the accretion of freshly synthesized Nrf2 and accompanying Nrf2 activation. It was proved that Nrf2 plays a non-negligible role in the adaptive response to cellular oxidative stress and other kinds of stress, and it may be a prospective target for the control of oxidative stress in AZS.

Qiangjing tablets are composed of a variety of traditional Chinese medicine, which could enhance renal function and improve sperm motility. It has been used in clinical practice for more than 20 years, which showed that certain curative effects on male infertility through a large number of experiments (Zhang et al., 2016b). Qiangjing tablets may enhance semen quality in azoospermic rats through mediating MAPK signaling pathway against oxidative stress (Li et al., 2018). Tripterygium glycosides may lead to infertility *via* regulating the Fas/FasL signaling pathway, decreasing sperm concentration, viability and vigor and inducing apoptosis in spermatogenic cells. Qiangjing tablets modified the reproductive function of male rats through reducing the level of the apoptotic factor FasL in testicular tissue (Zhang et al., 2016b). Also, Shenfu Qiangjing decoction diminish the liquefaction time of semen and ameliorate the weak testosterone level arising from all sorts of kidney-yang deficiency symptoms (Xiong et al., 2009).

However, to date, the therapeutic effect of Qiangjing tablets on weak spermatozoa is not known. Therefore, in this study, we investigated whether Qiangjing tablets could improve the reproductive function in azoospermia rats by activating the Nrf2/ARE pathway, which may provide a reference for the study of the pathophysiological basis of AZS.

## Material and Methods

### Animals and Groups

**FIGURE 1:**
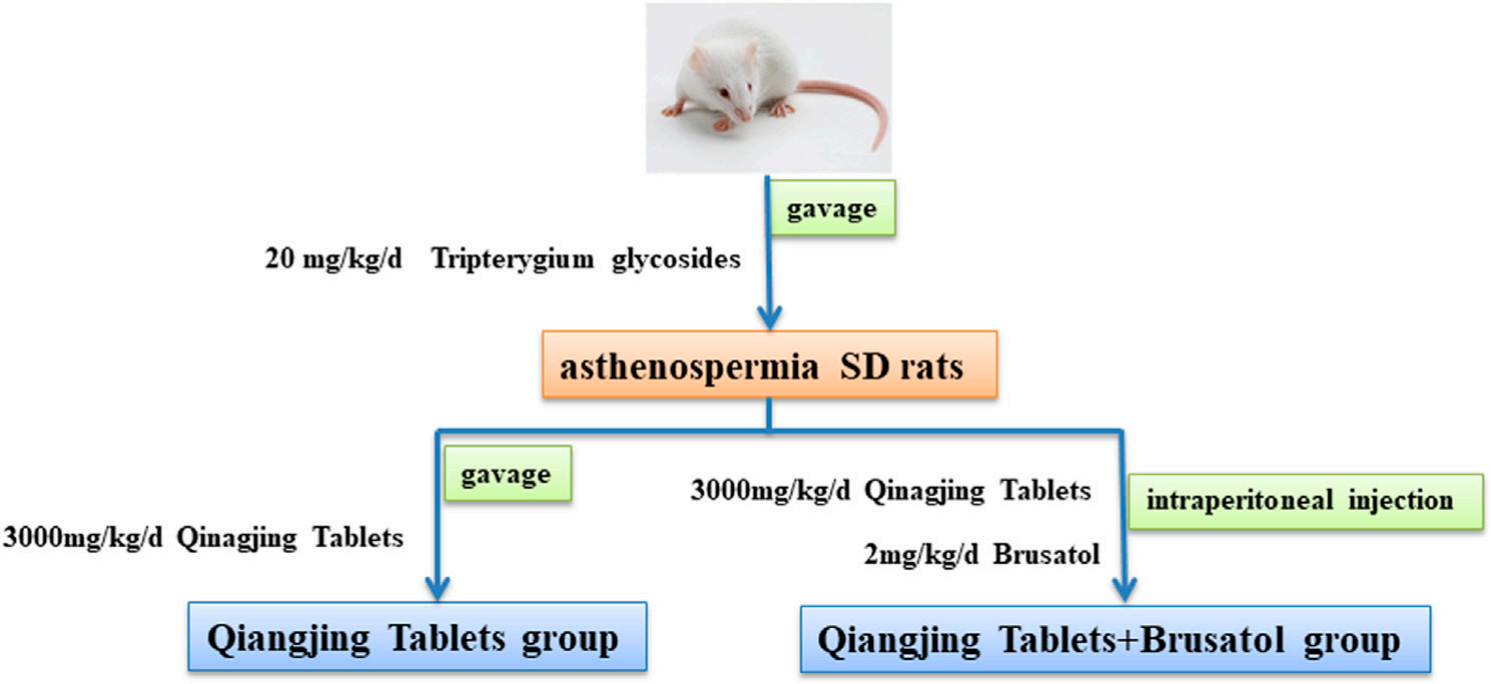
Schematic diagram of drug intervention. Construction of a male SD rat infertility model by intragastric administration of Tripterygium. Qiangjing Tablets and Brusatol were given in AZS rats.

Male SD rats (aged 2 months and weighed 300 ± 20 g) were purchased from Chengdu Dashuo biological Co., Ltd (Sichuan, China). Rats were kept in an isolated environment at 22–24°C and 40–60% humidity with a light/dark cycle for 12 h and provided with fresh drinking water and feed. Rats for AZS model were induced by gavage of 20 mg/ kg/ d Tripterygium glycosides solution for 3 weeks. Body weight of rats were recorded every week to adjust the dosage during the whole experiment. Then, AZS model rats were randomly divided into three groups (5 in each group). Specifically, model group, Qiangjing Tablets group (105 mg/kg/d, gavage for 2 weeks), Qiangjing Tablets + Brusatol group (2 mg/ kg/ d Brusatol, intraperitoneally injected for 2 weeks). Rats in blank group were given the same amount of saline solution by gavage. Subsequently, the rats were sacrificed after anesthesia. Then the epididymis was removed and the semen was taken for immediate testing of its quality (Figure 1). In this study, all animal experiments were conducted according to the ethical standards of experimental animals (Ethics committee approval document number: 20211304A).

### Reagents

Qiangjing Tablets is a Chinese Medicine Tablet. The specific formula is as follows: Ginseng Radix Et Rhizoma (Araliaceae; the root of Panax ginseng C. A. Meyer), Angelica Sinensis Radix (Umbelliferae; the dried root of Angelica sinensis (Oliv) Diels.), Rehmanniae Radix Praeparata (Scrophulariaceae; root tuber from Rehmannia glutinosa Libosch), Corni Fructus (Cornaceae; the pulp of the ripe fruit of Cornus oj-jZcinalis Sieb. etZucc.), Lycii Fructus (Solanaceae; the dried mature fruit of Lycium barbarum L.), Schisandrae Chinensis Fructus (Magnoliaceae; the dried ripe fruit of Schisandra chinensis (Turcz.) Baill.), Cuscutae Semen (Convolvulaceae; the dry mature seed of Cuscuta chinensis Lam), Plantaginis Semen (Plantaginaceae; the dried mature seeds of Plantago asiatica L. or Plantagodepressa Willd.), Epimedii Folium (Berberidaceae; the dried aerial parts of Epimedium Brevicornu Max-Im.), Common Curculigo orchioides (Amaryllidaceae; the rhizome of Curculigo orchioides Gaertn.), Herba Leonuri (Lamiaceae; the overground part of Leonurus japonicus Sweet). After appropriate grinding, they were extracted by reflux with 10 times and 8 times water respectively, 1 h each time, filtered, combined with the two kinds of filtrates, concentrated to 1 g/ ml under reduced pressure. Then methanol was added according to the volume ratio of 2:1 and left overnight. Take 100 ml supernatant, add methanol at 1:1, centrifugate at 3,000 r/ min for 3 min, and collect the supernatant. Take 1–5 ml supernatant and filtrate with 0.45 μm microporous membrane.

Reference solution: ferulic acid, Hyperoside, geniposide, loganin, verbascoside, morroniside, ginsenoside Re, Ginsenoside Rg1, schisandrin A, curculioside, stachydrine and motherwort alkaloid, and 0.1 mg of each Chinese medicine. Put them in a 10 ml volumetric flask, add methanol, and then use ultrasonic dissolution. Constant volume and obtain the mixed reference solution.

### Fingerprint Spectrum

Chromatographic conditions: chromatographic column was Ultimate LP-C18 column (250 mm × 4.6 mm, 5 μm), and the mobile phase was acetonitrile-0.05% phosphoric acid solution. The mobile phase ratio is shown in Table 1.

**TABLE 1:** Gradient elution conditions of acetonitrile −0.05% phosphoric acid.

Mobile phase		Time	Proportion
Acetonitrile	0.025% phosphate	0–5	5:95
Acetonitrile	0.025% phosphate	5–6	8:92
Acetonitrile	0.025% phosphate	6–10	10:90
Acetonitrile	0.025% phosphate	10–20	18:82
Acetonitrile	0.025% phosphate	20–23	20:80
Acetonitrile	0.025% phosphate	23–26	22:78
Acetonitrile	0.025% phosphate	26–30	25:75
Acetonitrile	0.025% phosphate	30–40	30:70
Acetonitrile	0.025% phosphate	40–50	45:55
Acetonitrile	0.025% phosphate	50–60	80:20
Acetonitrile	0.025% phosphate	60–75	90:10
Acetonitrile	0.025% phosphate	75–80	95:5

column temperature, 30°C; detection wavelength, 254 nm; Flow rate, 1 ml/min;injection volume, 10 μL.

### Sperm Parameters

After the last administration for 24 h, all rats were preoperatively anesthetized with 25% ethyl carbamate (4 ml/ kg, intraperitoneal injection), and then collected for the test. Bilateral epididymis were taken to measure sperm motility. The bilateral epididymis was put into Hams’F10 culture medium, which was pre-heated in the water bath box, and incubated at 37°C for 30 min until the epididymis sperm was completely dissociated. WLJY-9000 Weili Digital Color Sperm Quality Detection System was used to detect sperm motility. The sperm cells were obtained from the vas deferens and gently mixed to evaluate the kinematic parameters of sperm motility using the TOX IVOS sperm analyzer system (Hamilton Thorne Biosciences, Beverly, MA, United States), software version 12.21 [Cordero-Martínez et al., 2014].

### Hematoxylin and Eosin Staining

Testicular specimens were fixed with 10% paraformaldehyde for 24 h, paraffin embedded, sectioned for 4 μm, and stained with hematoxylin-eosin.

### TUNEL Staining

Following the typical scheme, sections were sequentially dewaxed, hydrated, and then put into dH2O. Immediately afterwards, these sections were cultivated in 20 μg/ ml proteinase K working solution for 15 min at ambient temperature. After that, the sections were removed, rinsed three degree with PBS, and placed in TUNEL reaction mixture at 37°C for 1 h. After rinsing three degree with PBS for 5 min, the slides were cultivated with HRP-streptavidin reagent (1:200) for 30 min at ambient temperature. After washing with PBS, sections were placed in a clean and airless place for natural air drying. The nuclei and seals were stained with antifade mount with DAPI (4′,6-Diamidino-2-Phenylindole, Dihydrochloride) (Thermo, D1306, 1:500) and SYTO™ nine Green Fluorescent Nucleic Acid Stain (Thermo, S34854, 1:500). The cells were cured in a dry environment at room temperature and protected from light for 24 h. The morphology of the cells was observed under the fluorescence microscope.

### Enzyme-Linked Immunosorbent Assay

After 5 weeks of administration, fasting for 12 h, the blood was collected from the ocular fundus venous plexus. The blood was stood at 4°C for 2 h, and was centrifuged at 3,500 r/ min twice with 10 min each time. The levels of Estradiol, testosterone, luteinizing hormone, SOD, GSH-Px, GSH, and MDA were quantified by ELISA kits following the manufacturer’s instructions. They were purchased from Jiancheng Institute of Biotechnology (Nanjing, China).

### Western Blot

Western blotting was performed as previously reported. Proteins were extracted with RIPA Lysis Buffer (Abcam, Ab156034, United Kingdom) and their concentrations were assayed with Pierce™ BCA Protein Assay Kit (Thermo). Protein extracts were transferred onto polyvinylidene fluoride membranes by using SDS-PAGE. Membranes were then closed with Tris-buffered saline containing 5% skim milk powder, which were cultivated at 4°C overnight with primary antibodies against p-Nrf2 (ab31163; 1:1,000; Abcam; United States), Nrf2 (ab137550; 1:1,000; Abcam; United States), HO-1 (ab1324; 1:250; Abcam; United States) and Keap-1 (ab132384; 1:1,000; Abcam; United States). Subsequently, a secondary Goat Anti-Rabbit IgG (HRP) antibody (Abcam, ab205718, 1:2000) was applied to incubate with membranes at room temperature for 1 h. The blots were assayed by using the Novex™ ECL Chemiluminescent Substrate Reagent Kit (Thermo Fisher Scientific™, WP20005, United States). β-actin was used as an internal reference protein. The results were analyzed with ImageJ software.

### Flow Cytometry

The semen samples were washed twice with PBS, centrifuged (3000 r × 5 min), and then suspended and precipitated with PBS. The sperm density was adjusted to 5 × 106/ ml. 100 μL sperm suspension mixed with 2 μL Annexin V-FITC and 5 μL PI was added to 500 μL Binding Buffer. After incubation at room temperature and in the dark for 15min, flow cytometry was used to detect 10,000 sperm in each sample. AV + /PI- were early apoptotic sperm, AV-/PI- was normal living sperm, and PI + was dead sperm. Apoptosis was analyzed by SPSS software, and preprocessed Q1-LR data was selected for single-factor statistical analysis.

### JC-1 Assay

Sperm suspensions (2x10/ ml) were incubated with 10 μg/ ml JC-1 dye (20 min, 37°C, dark). Fluorescence was generated on an Axio Imager M2 microscope (Carl Zeiss), and green JC-1 monomers (Ex/En 490/529, loss of AYm) and orange JC-1 aggregates (Ex/Em 514/590, normal AYm) were viewed simultaneously. The mitochondria uncoupler carbonyl cyanine p-(trifluoromethoxy) phenylhydrazone (FCCP) served as the negative control. For each individual 1,000 sperm cells were counted.

### CCK-8 Assay

GC-1 spgs were collected from the American Type Culture Collection (Manassas, VA, United States) and incubated in complete Dulbecco’s modified Eagle medium (DMEM, Gibco, Grand Island, NY, United States) containing 10% fetal bovine serum (FBS, Gibco) with 100 units/ mL penicillin and 100 μg/ ml streptomycin (Sigma, St-Louis, MO, United States) in a humidified air including 5% CO2 at 37°C. When cells density reached 5 × 103 cells/ mL (37°C, 12 h), which were considered control group. The cells were divided into five groups (Moore and Reijo-Pera, 2000): Blank group (Hwang et al., 2011); Model group, cells were stimulated by H2O2 for 3 days (Liu et al., 2015); Qiangjing Tablets group (50 μM/ ml) (Balkan et al., 2008); Qiangjing Tablets + Brusatol groups (25 μM/ ml Brusatol). GC-1 spgs were collected after 48 h, analyzed by Cell Counting Kit-8 (CCK-8) according to the instructions of the manufacturer (Beyotime, Shanghai, China).

### Statistical Analysis

The SPSS 17.0 statistical software and GraphPad Prism seven software were used for statistical analysis of the experimental data. All the datas were indicated as mean ± standard deviation (mean ± SD). One-way ANOVA (one-way analysis of variance), was used for comparisons between more groups, and LSD-*t* test (Least significant digital) was used for homogeneity of variance, and Dunnett-*t* test was used for heterogeneity of variance. *p*-values < 0.05 were considered to be significant.

## Results

### Fingerprint Spectrum Analysis of Qiangjing Tablets

**FIGURE 2:**
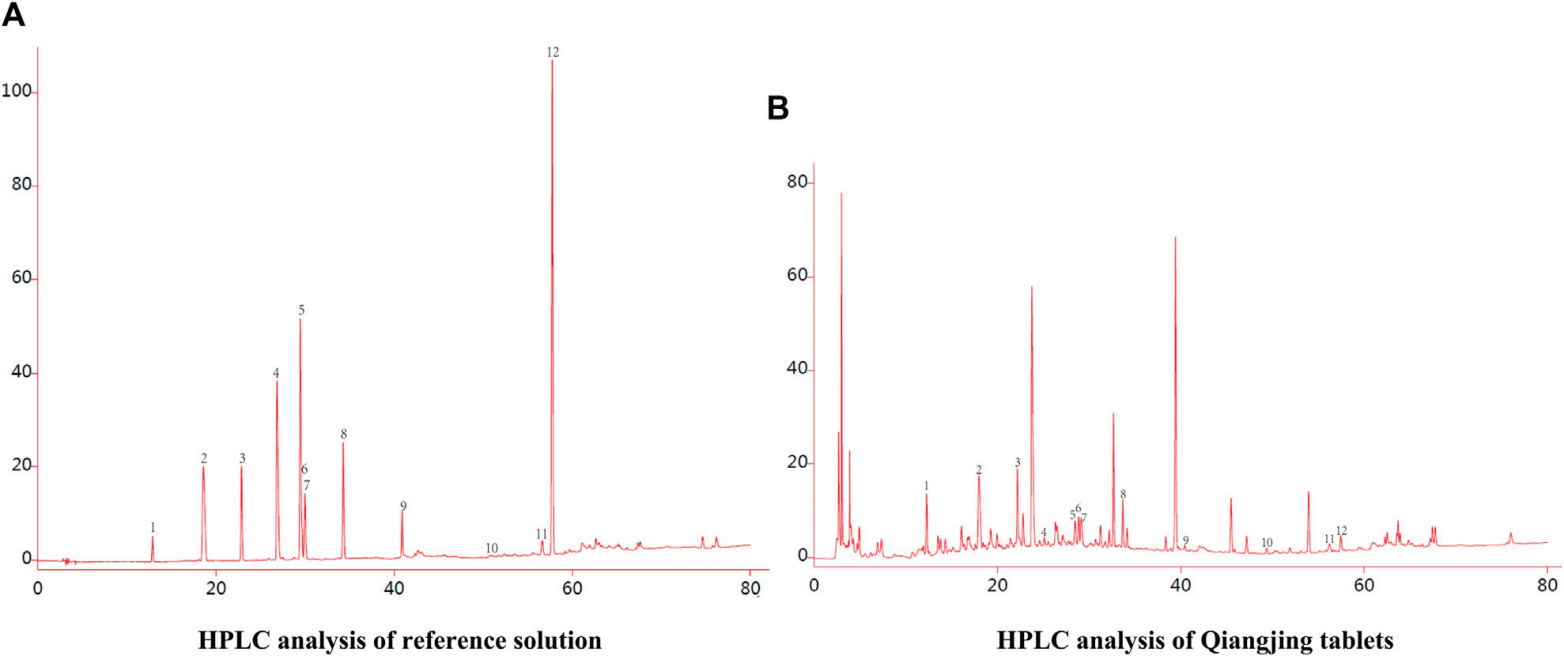
Fingerprint spectrum analysis of Qiangjing tablets. **(A)** HPLC analysis of reference solution. **(B)** HPLC analysis of Qiangjing tablets. (1. Geniposide; 2. Mononoside; 3. Loganin;. 4. Motherwort alkaloid; 5. Mullein glycoside; 6. Hypericin; 7. Ferulic acid; 8. Xanthoside; 9. Icariin; 10. Schisandrin; 11. Ginsenoside Rg1; 12. Ginsenoside Re).

In order to provide a reliable method for the quality control of Qiangjing Tablets, fingerprints of Qiangjing tablets and reference solution were compared. At the same chromatographic conditions, there were 12 common peaks in fingerprints of both Qiangjing tablets and reference solution, which showed that the type or content of Qiangjing tablets appeared to no change greatly before and after preparation (Figure 2).

### Effect of Qiangjing Tablets on Sperm Parameters in AZS Rats

**FIGURE 3:**
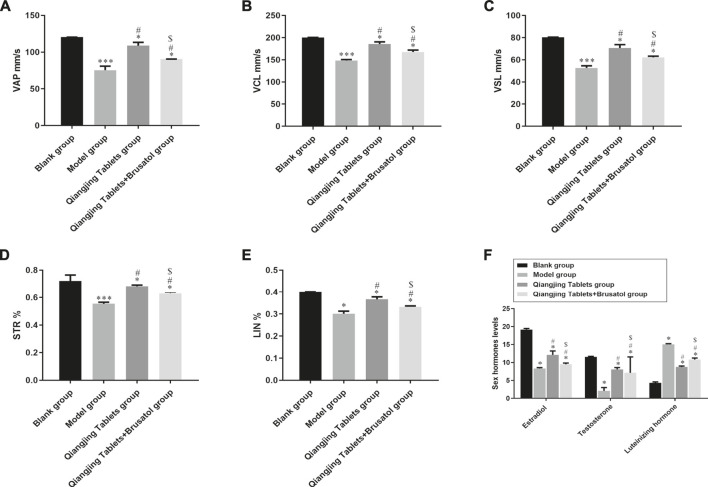
Effect of Qiangjing Tablets on sperm parameters in AZS rats. **(A)** velocity average path (VAP, mm/s). **(B)** velocity curved line (VCL, mm/s). **(C)** velocity straight line (VSL, mm/s). **(D)** straightness (STR, %). **(E)** linearity (LIN, %). **(F)** Expression levels of sex hormone (Estradiol, Testosterone and Luteinizing hormone) in Blank group, model group, Qiangjing Tablets group and Qiangjing Tablets + Brusatol group. Values are expressed as mean ± SD, *n* = 15 per group. *p < 0.05 *vs.* Blank group; ^**#**^ *p* < 0.05 *vs.* model group; ^**$**^ *p* < 0.05 *vs.* Qiangjing Tablets group.

In order to evaluate Qiangjing tablets whether improve sperm quality through detect sperm motility, sperm concentration, sperm viability and kinematic parameters of sperm and to investigate the effect of Qiangjing Tablets on sex hormone secretion by ELISA. Table 2 displayed that the sperm motility, sperm concentration and sperm viability in the model group were significantly decreased as compared with Blank group, and were significantly increased as compared with Qiangjing Tablets group. This suggested that Qiangjing Tablets significantly increased semen quality in rats. Compared with Qiangjing Tablets group, sperm motility, sperm concentration and sperm viability were dramatically reduced in Qiangjing Tablets + Brusatol group (Table 2). This was also true for changes in kinematic parameters of sperm (VAP, VCL, VSL, STR, and LIN) (Figures 3A–E). The expression levels of Estradiol and testosterone were significantly decreased in the model group, Qiangjing Tablets group and Qiangjing Tablets + Brusatol group than Blank group, were significantly increased in Qiangjing Tablets group and Qiangjing Tablets + Brusatol group than model group, were significantly reduced in Qiangjing Tablets + Brusatol group than Qiangjing Tablets group. The expression level of luteinizing hormone was opposite to that of Estradiol and testosterone (Figure 3F).

**TABLE 2:** Sperm motility, sperm concentration, and sperm viability of different groups after the treatment of Qiangjing Tablets.

Group	Sperm motility (%)	Sperm concentration (× 10^6^/ ml)	Sperm viability (%)
Blank	75.31 ± 2.12	148.71 ± 8.57	86.92 ± 3.23
Model	42.88 ± 2.97^a^	63.88 ± 2.53^a^	54.42 ± 1.54^a^
Qiangjing Tablets	57 ± 1.19^a^ ^,^ ^b^	127.15 ± 2.43^a^ ^,^ ^b^	71.15 ± 1.11^a^ ^,^ ^b^
Qiangjing Tablets + Brusatol	48.06 ± 1.56^a^ ^,^ ^c^	97.24 ± 5.13^a^ ^,^ ^c^	60.04 ± 1.04^a^ ^,^ ^b^ ^,^ ^c^

Values are expressed as mean ± SD, *n* = 15 per group.

a p < 0.05 *vs*. Blank group.

b p < 0.05 *vs*. model group.

c p < 0.05 *vs*. Qiangjing Tablets group.

### Effect on Morphology in Testis Induced by Qiangjing Tablets in AZS Rats

**FIGURE 4:**
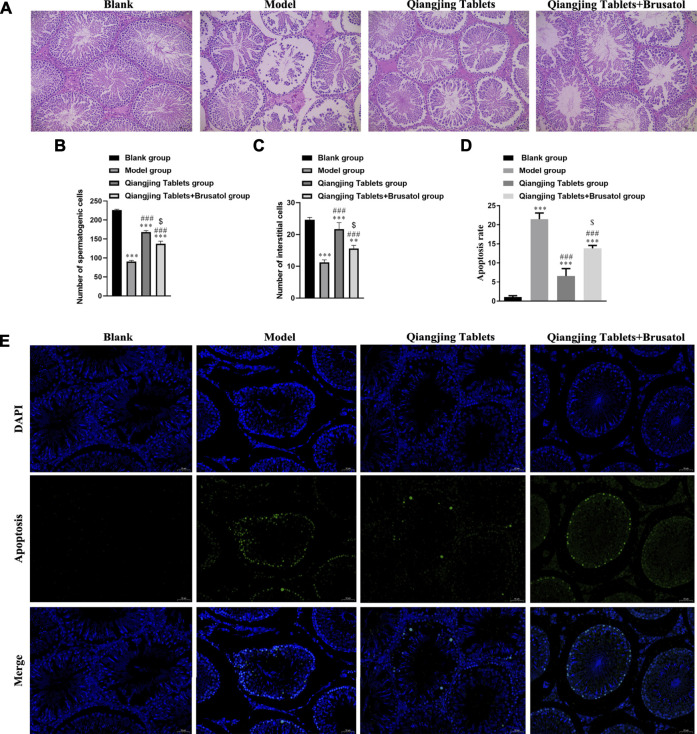
Effect on morphology in testis induced by Qiangjing Tablets in AZS rats. **(A–C)** H and E staining to show histopathological changes in the testis in Blank, model, Qiangjing Tablets and Qiangjing Tablets + Brusatol groups (original magnifcation × 200; scale bar, 10 μm). Red: spermatogenic cells: green: interstitial cells: blue: seminiferous tubule. **(D–E)** Green is apoptotic cells by Tunel in Blank, model, Qiangjing Tablets and Qiangjing Tablets + Brusatol groups (original magnification, 400 × ). Values are expressed as mean ± SD, *n* = 15 per group. **p < 0.01 *vs*. Blank group; ***p < 0.001 *vs* Blank group; ##*p* < 0.01 VS model group; ###*p* < 0.001 *vs* model group; ^$^ *p* < 0.05 *vs* Qiangjing Tablets group.

We studied the effect of Qiangjing tablets on testicular function by HE staining and Tunel. Pathological changes of testis were observed by H and E staining. There were no obvious pathological changes in the testis in Blank group. In model group, the spermatogenic tubule space was increased, the amount of interstitial cells, spermatogenic cells, and sperms in the seminiferous tubules were markedly reduced. However, after intervention with Qiangjing Tablets, the above changes improved significantly. The spermatogenic cells was arranged more orderly, compact and normal in morphology and structure, and the quantity of sperms, spermatogenic cells and interstitial cells was remarkably increased. Nonetheless, Brusatol reversed these improvements (Figures 4A–C). We investigated whether Qiangjing Tablets inhibited apoptosis by TUNEL staining in spermatogenic cells. The apoptosis rate was significantly increased in model group, Qiangjing Tablets group and Qiangjing Tablets + Brusatol group than Blank group, was significantly reduced in Qiangjing Tablets group and Qiangjing Tablets + Brusatol group than model group, and was significantly reduced in Qiangjing Tablets + Brusatol group than Qiangjing Tablets group (Figures 4D,E).

### Effect on Cell Apoptosis of Testis and Viability of GC-1 Spgs

**FIGURE 5:**
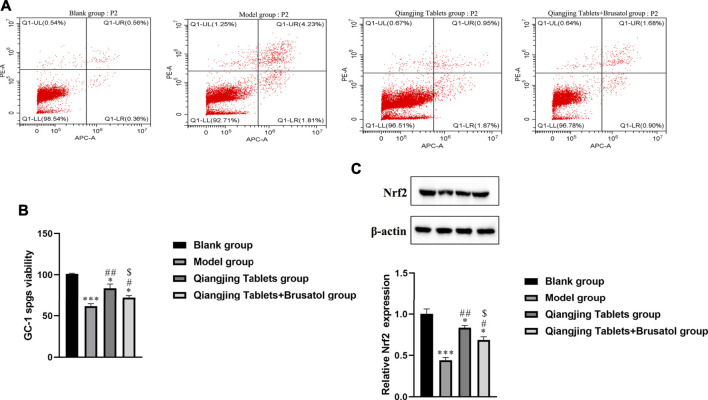
Effect on cell apoptosis of testis and viability of GC-1 spgs**. (A)** Flow cytometry for Early + Late apoptosis in Blank, model, Qiangjing Tablets and Qiangjing Tablets + Brusatol groups. **(B)** CCK-8 assay for assay viability. **(C)** Western blotting for Nrf2 protein. Values are expressed as mean ± SD, *n* = 15 per group. *p < 0.05 *vs*. Blank group; ***p < 0.001 *vs*. Blank group; #*p* < 0.05 *vs*. model group; ##*p* < 0.01 *vs*. model group; ^$^ *p* < 0.05 *vs*. Qiangjing Tablets group.

It is well known that intracellular oxidative stress and mitochondrial dysfunction lead to cellular damage and neurotoxicity (Ciccone et al., 2013). Hence, flow cytometry was used to detect apoptosis in each group. Apoptosis was analyzed by flow cytometry, which was investigated the possible mechanism of apoptosis inhibited by Qiangjing Tablets. Compared with the model group, Qiangjing tablets could inhibit early + late apoptosis (Figure 5A; Table 3). GC-1spgs viability of different groups was then determined by the CKK-8 method. The results showed that the proliferation of GC-1 spgs was dramatically increased in Qiangjing Tablets group compared with model group (Figure 5B). The proliferation of GC-1 spgs was significantly decreased in Qiangjing Tablets + Brusatol group than Qiangjing Tablets group. Western blot for Nrf2 protein expression in diffierent GC-1 spgs groups. The results showed that Nrf2 protein was markedly increased in Qiangjing Tablets group compared with model group. Nrf2 expression was significantly decreased in Qiangjing Tablets + Brusatol group than Qiangjing Tablets group because Brusatol is a specific the Nrf2 pathway (Figure 5C).

**TABLE 3:** Effects on apoptosis induced by Qiangjing Tablets ($\bar{x}$ ± SD).

Group	N	Early apoptosis analysis %	Early + Late apoptosis %
Blank	15	0.32 ± 0.12	0.92 ± 0.10
Model	15	1.88 ± 0.63^a^	5.10 ± 0.86^a^
Qiangjing Tablets	15	1.84 ± 0.28	2.81 ± 0.52^b^
Qiangjing Tablets + Brusatol	15	1.87 ± 0.57	3.16 ± 0.68^b^

Values are expressed as mean ± SD, *n* = 15 per group.

a p < 0.05 *vs.* Blank group.

b p < 0.05 *vs*. model group.

### Effect of Qiangjing Tablets on Mitochondrial Damage and Oxidative Stress of Sperm in AZS Rats

**FIGURE 6:**
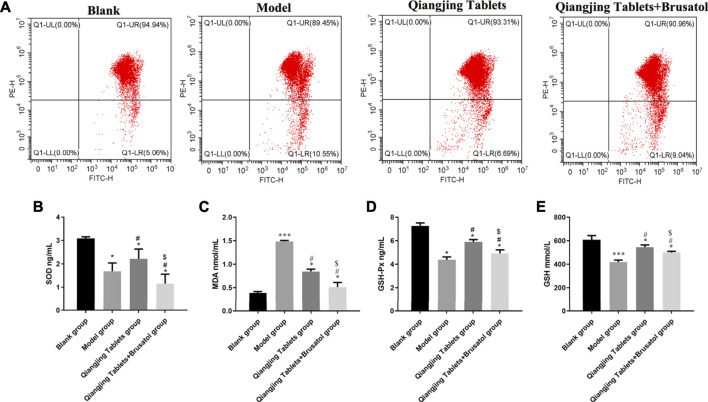
Effect of Qiangjing Tablets on mitochondrial damage and oxidative stress of sperm in AZS rats. **(A)** JC-1 for mitochondrial damage in Blank, model, Qiangjing Tablets, and Qiangjing Tablets + Brusatol groups. **(B–E)** ELISA for activity SOD and GSH-Px, and content of MDA and GSH in testis. Values are expressed as mean ± SD, *n* = 15 per group. *p < 0.05 *vs.* Blank group; ***p < 0.001 *vs.* Blank group; #*p* < 0.05 *vs.* model group; ^$^ *p* < 0.05 *vs.* Qiangjing Tablets group.

Oxidative stress plays an essential role in mitochondrial damage, which was assessed via estimating content MDA and GSH and activity of SOD and GSH-Px by ELISA in the study. Change of mitochondrial membrane potential was detected by JC-1. The PE/FITC ratio was notably increased in model group than Blank group, and was significantly decreased in Qiangjing Tablets group and Qiangjing Tablets + Brusatol group than model group (Figure 6A). The results show that the activity SOD and GSH-Px, GSH content were strikingly reduced in model group. After Qiangjing tablet treatment, SOD and GSH PX activities and GSH content increased significantly. However, Brusatol reversed these changes. The change of MDA content is opposite to that of SOD, GSH-Px and GSH in Blank group, model group and Qiangjing Tablets group (Figures 6B–E).

### Effect of Qiangjing Tablets on Nrf2/ARE Pathway in AZS Rats

**FIGURE 7:**
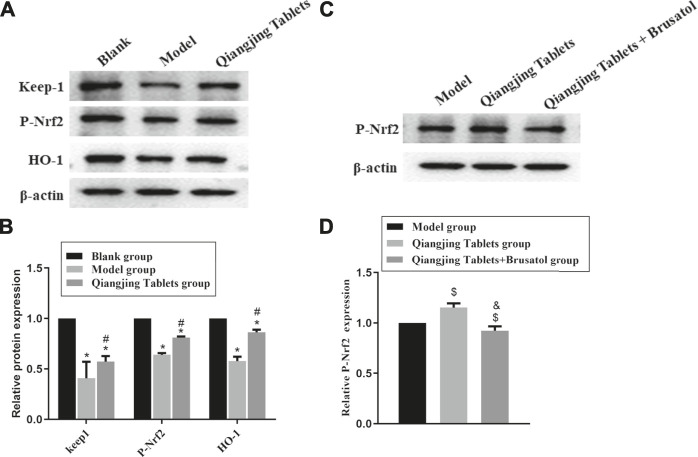
Effect of Qiangjing Tablets on Nrf2/ARE pathway in AZS rats. **(A)** Western blotting was used to assay the expression of P-Nrf2, Keap1 and HO-1 in Blank, Model and Qiangjing Tablets groups. **(B)** Optical density values of P-Nrf2, Keap1 and HO-1 were quantified and analyzed in each group. **(C)** Western blotting was applied to detect the expression of P-Nrf2 in Model, Qiangjing Tablets and Qiangjing Tablets + Brusatol groups. **(D)** Optical density values of P-Nrf2 was quantified and analyzed in each group. Values are expressed as mean ± SD, *n* = 15 per group. *p < 0.05 *vs.* Blank group; ^**#**^ *p* < 0.05 *vs.* model group; ^$^ *p* < 0.05 *vs.* model group; ^&^ *p* < 0.05 *vs.* Qiangjing Tablets group.

To demonstrate that activation of Nrf2/ARE signaling pathway is the primary mechanism by which Qiangjing Tablets exert its therapeutic effects with promoting reproductive function in SD rats with AZS, we analyzed HO-1, Keap1 and P-Nrf2 protein levels by Western bolting. Compared with the Blank group, HO-1, Keap1 and P-Nrf2 in model group and Qiangjing Tablets group were strikingly reduced. Compared with the model group, HO-1, Keap1 and P-Nrf2 in Qiangjing Tablets group were significantly increased (Figures 7A,B). This indicated that Qiangjing tablet could activate Nrf2/ARE pathway. Brusatol is a particular suppressor of the Nrf2 pathway. P-Nrf2 level was significantly decreased in Qiangjing Tablets + Brusatol group than Qiangjing Tablets group (Figures 7C,D).

## Discussion

In male infertility patients, AZS accounts for about 19%, which is one of the important factors leading to male infertility (Shay et al., 2016). In recent years, the research direction of male infertility treatment focuses on microsurgery and assisted reproductive technology. Although there are many kinds of drug treatment, none of them has been recognized in the world due to the uncertain curative effect. Traditional Chinese medicine to treat male infertility has a very old history, which can significantly improve sperm motility, but its mechanism has not been clarified. In this study, Qiangjing Tablets was identified to enhance reproductive function in AZS rats by activating Keap1/Nrf2 pathway, inhibiting mitochondrial damage, resisting oxidation and enhancing sperm motility.

Semen quality, as a major factor affecting male fertility, is not only closely related to sperm motility, but also related to sperm concentration and proportion of normal sperm morphology (Pan and Huang, 2015). Recent studies have found that taurine may protect epididymal epithelium structure, improve spermsecretion activity (Du et al., 2019). In the study, it was found that Qiangjing Tablets had a significant ameliorative effect on the lesions in the testes of AZS rats, as evidenced by an increase in the amount of spermatogonia cells, interstitial cells and mature spermatozoa, and normalization of the structure and reduction of the gap of the seminiferous tubules. The results indicated that Qiangjing Tablets could effectively inhibit morphologic changes in sperm. Intracellular ROS are thought to originate mainly from mitochondria. Sperm mainly used ATP produced by mitochondrial aerobic oxidation to maintain its athletic ability (Eddy et al., 2003). However, redundant ROS may lead to oxidative stress and mitochondrial dysfunction, which in turn impair semen quality and structural integrity of sperm (Sikka, 2001). Studies have demonstrated that lower sperm motility is mainly associated with mitochondrial dysfunction (Nowicka-Bauer et al., 2018). The decrease of mitochondrial membrane potential is a marker event of apoptosis. Interestingly, taurine improves sperm quality and function by enhancing mitochondrial energy metabolism epididymal antioxidant ability of AZS rats (Du et al., 2019). The study suggested that PE/FITC ratio reduced, which was shown to Qiangjing Tablets inhibit apoptosis. The changes of MDA content and SOD activity in testis tissue were tested, which confirmed the occurrence of oxidative stress. Oxidative stress is an essential role in the damage of blood testis barrier. After treatment with Qiangjing Tablets, SOD, GSH-Px and GSH upregulated and MDA downregulated, suggesting that Qiangjing Tablets could resist the oxidative damage.

**FIGURE 8:**
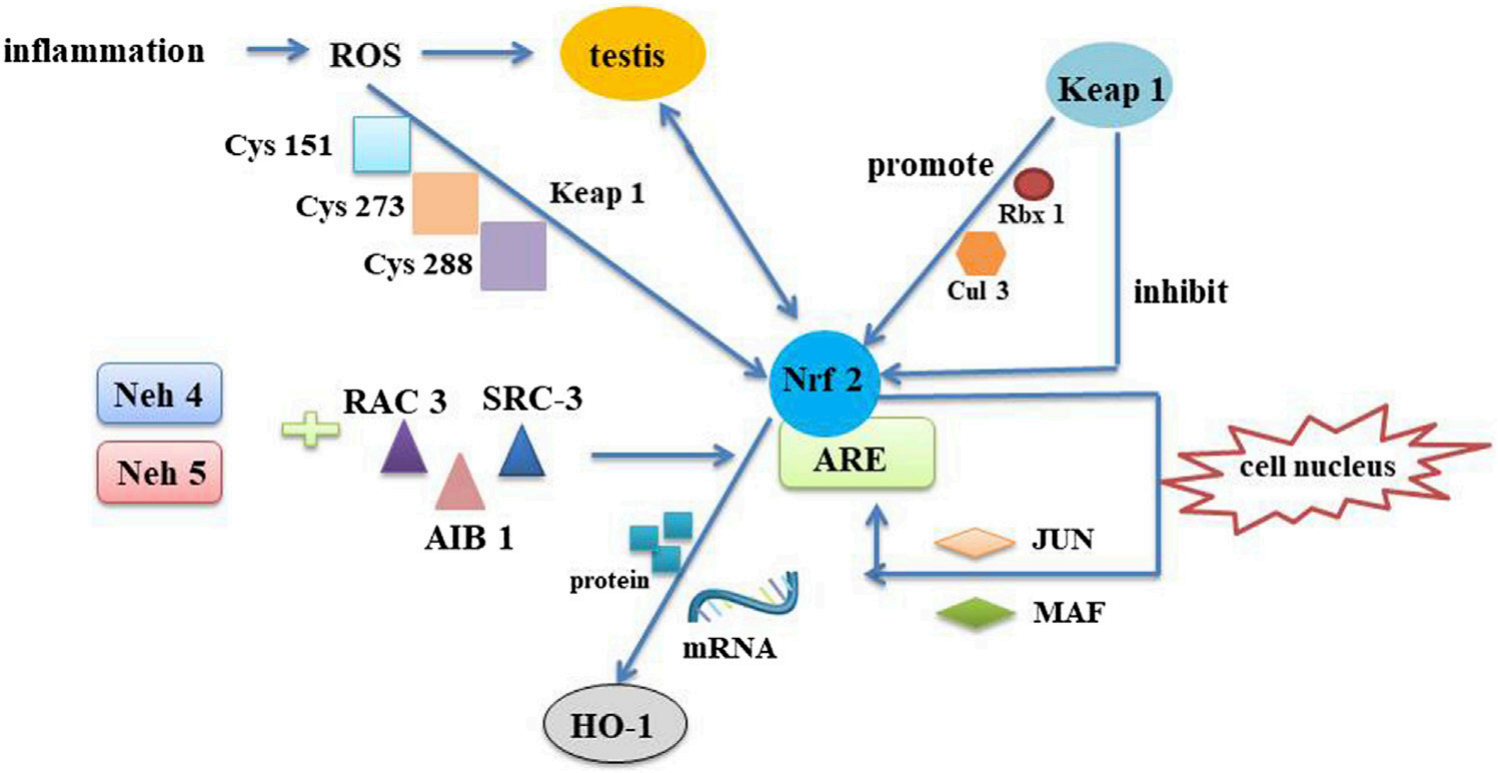
Mechanism of Nrf2/ARE pathway. The Nrf2/ARE pathway is an intrinsic mechanism of resistance to oxidative stress. In the pathological inflammatory process, immune cells are first waked. These cells are then recruited to the injury site and produce ROS to destroy the testis. Neh4 and Neh5, a part of functional domains of Nrf2, mutual effect with nuclear cofactor RAC3/AIB1/SRC-3 can result in expression intensity of Nrf2-targeted ARE gene. When oxidative stress occurs, ROS respond to cysteines on Keap1 to induce a conformational change in the release of Nrf2, thereby eliminating its degradation. As a consequence, Keap1-assisted Cullin3/Rbx1-dependent polyubiquitination of Nrf2 is interdicted, and Nrf2 is released and quickly relocated into the nucleus. Once entering the nucleus, Nrf2 is engaged in heterodimerization with minor MAF or JUN proteins and their complexes combine with the ARE. HO-1 is the derivable isomer and limiting velocity enzymes. Nrf2 upregulats mRNA and protein expression through induction of HO-1 gene. Nrf2, Nuclear factor erythroid 2-related factor 2; Keap1, Kelch-like ECH-associated protein 1; ARE, antioxidant response element; MAF, musculoaponeurotic fibrosarcoma; RAC3, receptor-associated coactivator 3; SRC-3, steroid receptor coactivator-3; Neh5, Nrf2-ECH homology 5; Neh4, Nrf2-ECH homology 4; ROS, reactive oxygen species; Rbx1, RING-box protein 1; HO-1, heme oxygenase-1.

The Nrf2/ARE signaling pathway is an endogenous antioxidant system whose major function is to regulate cytotoxicity and redox conditions (Chen et al., 2012) (Figure 8). Here’s the interesting part, Nrf2 is a basic leucine zipper (bZIP) transcription activators that mediates the expression of more than 100 oxidative stress-related genes (Niture et al., 2010). It has been shown that Nrf2 inhibits the inflammatory response by regulating the expression of phase II detoxification enzymes (NADPH, glutathione peroxidase, heme oxygenase-1 (HO-1) and antioxidant genes) thereby defending cells from various types of damage (Braun et al., 2002; Chen et al., 2006; Arisawa et al., 2007). Thus, Therefore, Nrf2 could be explored as a target to inhibit oxidative stress and inflammation. It has been demonstrated that sperm production is disrupted in mice with knockout of transcription factor Nrf2. In heat stress-induced mice, Nrf2 inhibits testicular oxidative damage (Li et al., 2013). Of them, HO-1 is a representative one of the stress-responsive enzymes that upregulate Nrf2, exhibiting significant anti-inflammatory and antioxidant properties (Ahmed et al., 2017). Studies have shown that Naringenin decreases oxidative stress and ameliorates mitochondrial dysfunction by modulating the Nrf2/ARE pathway in neurons (Wang et al., 2017). The results demonstrated strong increases of HO-1, Keap1 and P-NRF2 protein levels in Qiangjing Tablets group compared with model group. Brusatol is a specific inhibitor of the Nrf2 pathway (Nakamura et al., 2010). P-Nrf2 level was significantly decreased in Qiangjing Tablets + Brusatol group. Our study suggested that Qiangjing Tablets facilitated viability of GC-1 spgs, and ulteriorly promoted reproductive function of AZS rats by Keap/Nrf2 pathway.

## Conclusion

In summary, Qiangjing Tablets could inhibit spermatogenic apoptosis and oxidative stress, upregulate male sex hormone to improve the reproductive function in AZS rats via regulating Keap/Nrf2 pathway.

## Data Availability

The raw data supporting the conclusions of this article will be made available by the authors, without undue reservation.
